# A Randomized Control Trial Of Anti-Inflammatory Regional Hypothermia On Urinary Continence During Robot-Assisted Radical Prostatectomy

**DOI:** 10.1038/s41598-018-34657-4

**Published:** 2018-11-05

**Authors:** Linda M. Huynh, Douglas Skarecky, James Porter, Christian Wagner, Jorn Witt, Timothy Wilson, Clayton Lau, Thomas E. Ahlering

**Affiliations:** 1 Department of Urology, University of California, Irvine, Orange, CA USA; 2 Swedish Urology Group, Swedish Medical Center Seattle and Issaquah, Seattle, WA USA; 3 Urology Department, St. Antonius-Hospital Gronau GmbH, Westfalen, Germany; 4 Department of Urology, City of Hope National Medical Center, Duarte, CA USA

## Abstract

The present study seeks to present a single-blind, randomized control trial of a hypothermic anti-inflammatory device, the endorectal cooling balloon (ECB), to assess whether regional hypothermia could improve 90-day and time to pad-free continence following robot-assisted radical prostatectomy (RARP). Five high-volume surgeons at three institutions had patients randomized (1:1) to regional hypothermia with ECB versus control. Patients were blinded to device use, as it was inserted and removed intraoperatively. Knowledge of device use was restricted to the operating room personnel only; recovery room and ward nursing staff were not informed of device use and instructed to indicate such if a patient inquired. An independent and blinded data acquisition contractor assessed outcomes via components of the EPIC and IPSS. The primary outcome was categorical pad-free continence at 90-days and the secondary outcome was a Kaplan-Meier time-to pad-free continence at 90 days. 100 hypothermia and 99 control patients were included. The primary outcome of 90-day pad-free continence was 50.0% (27.8–70.0%) in the hypothermia group versus 59.2% (33.3–78.6%) in the control (p = 0.194). The secondary outcome of Kaplan Meier analysis for time to 90-day continence was not statistically significant. At one year, there were also no statistically significant differences in continence recovery. Post-hoc analysis revealed a trend towards improvement in continence in one of three sites. Overall, the trial demonstrated no benefit to regional hypothermia either in our primary or secondary outcomes. It is suggested that surgical technique and prevention of surgical trauma may be more advantageous to improving continence recovery.

## Introduction

The short and long-term functional effects of incontinence and erectile dysfunction following robot-assisted radical prostatectomy (RARP) are well known, and were integrally weighed in the United States Preventative Services Task Force evaluation of prostate specific antigen-(PSA) based prostate cancer screening^1,2^. Post-prostatectomy recovery of urinary continence, preferably defined as pad-free continence, is noted for diverse outcomes reported in numerous studies over the past two decades^3–8^. Recent meta-analysis comparing open and robotic approaches showed improved continence with RARP^9^. However, in order to improve continence, the etiology of post-RARP incontinence must be logically and systematically addressed. Incontinence is known to be multifactorial, but it remains to be demonstrated whether inflammation or technique and surgeon skill play the major role^5^.

As suggested above, technique and surgeon skill is well established; but, a comparison of the impact of patient-related factors such as age, co-morbidity status, prostate size, etc. remains a question. Most single surgeon studies report age as an independent factor for delaying time to continence and overall continence^9–11^. It is our belief that the impact of age is related to inflammation as inflammation in this setting plays a major potential role as individual surgeon technique and operative time are not shown to vary due to age. Hence, our presumption is that as men get older they do not recover from the same amount of trauma (i.e. inflammation) of surgery as well as if they were 10–20 years younger. Indeed, inflammation and acute injury secondary to dissection, traction, and thermal injury are linked to damage and transient dysfunction of muscle and neural networks have been shown to be mitigated with preemptive hypothermia prior to surgical dissection^12–18^.

**Figure 1:**
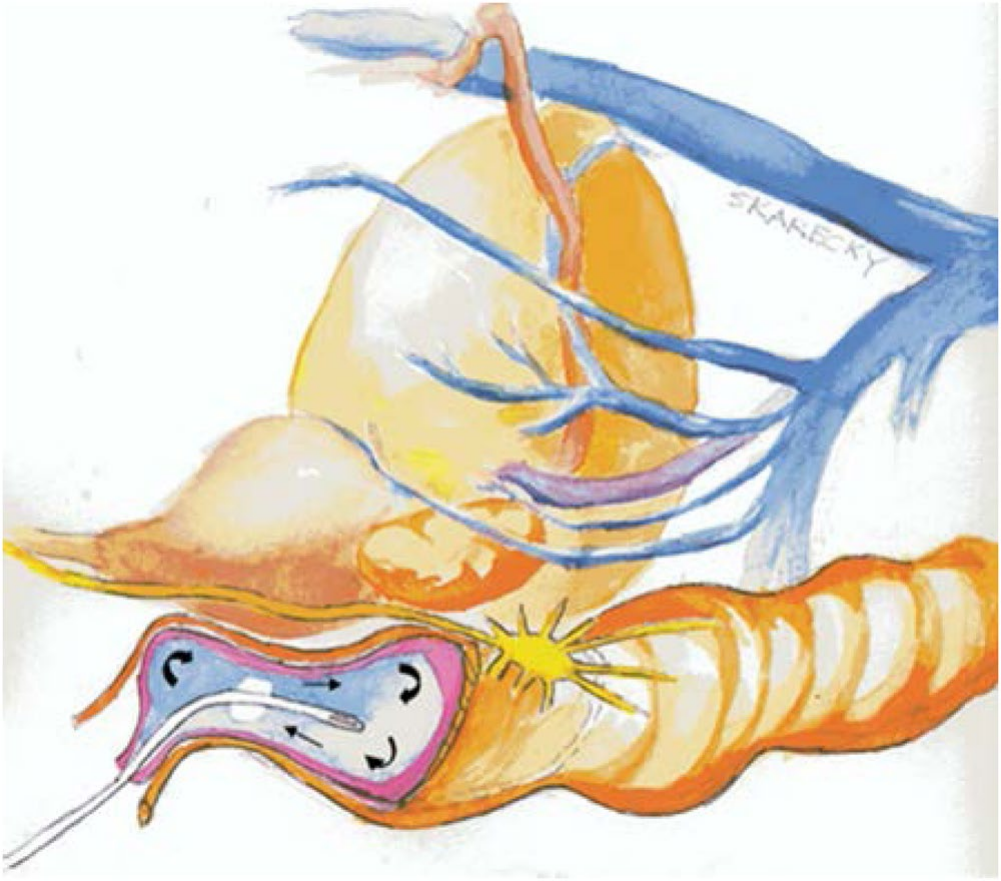
Schematic depicting endorectal cooling balloon (ECB).

In 2009, our group developed and patented technology of an endorectal cooling balloon (ECB) which circulates 4 degrees Celsius saline to provide regional hypothermia during RARP in an effort to reduce post-surgical inflammation (Fig. 1)^19^. We demonstrated that tissues in multiple areas in the surgical field had temperatures of approximately 25 degrees Celsius with our device. Further, upon pathological review of post RARP prostatectomy specimens there was a 50% reduction (p = 0.008) in neutrophil cells in tissue exposed to hypothermia during RARP^20^. Our single surgeon studies showed a significant reduction in time to continence (39 vs. 62 days, p = 0.0003) in patients who received regional hypothermia and improved overall long-term continence at one year (94% vs 86%, P = < 0.001), particularly in patients over the age of 70^4,20^. Herein, we conducted a randomized, single-blinded, controlled trial to assess whether regional hypothermia with the ECB device during RARP could improve time to and overall urinary continence.

## Methods

### Study design

Patients who were candidates for robot-assisted radical prostatectomy (RARP) for the treatment of prostate cancer were recruited to the trial. Exclusion criteria included baseline or history of urinary incontinence, patients who did not speak or understand English (United States sites only), prior extensive pelvic surgery, history of prior treatment of prostate cancer, prior intra-operative injuries, inadequate hemostasis, and/or serious concurrent medical conditions likely to result in death during the next 12 months.

All patients provided informed consent to participate in a phase 3, single-blind, randomized clinical trial of return to continence within 90 days after RARP with or without use of an ECB to produce regional hypothermia. This trial was planned with intent to obtain FDA approval of the ECB, as patented by the University of California in 2009 and licensed to study and potentially market to Philips Healthcare Inc. Three external high volume centers (two from USA and one in Europe) were invited to contribute a total of five surgeons. Surgeons had between 500 and 3,500 case experience and each performed greater than 100 procedures per year. The principle investigator (TA) was not included in the study and traveled to each center to train, advise, and confirm proper use of the ECB during RARP.

The institutional review board of each center approved the study (Ethik-Kommission der Ärztekammer Westfalen-Lippe und der Westfälischen Wilhelms-Universität Münster [CG, JW], City of Hope National Medical Institutional Review Board [TW, CL] and Western Institutional Review Board [JP]), compliance with the Health Insurance Portability and Accountability Act was maintained, and the trial was formally registered as a randomized control trial (NCT01920035). From May through October 2013, 199 patients who were recruited, consented, and planned to undergo an RARP underwent explicit randomization into two parallel groups (CONTROL or TREATMENT) by centralized computer-generated software and assigned to intervention by the surgeons at each study site. To ensure balance by study site, a stratified and block randomization algorithm was used and randomization was stratified by study site and block sizes of six were used within each strata. Cross-over between patient assignments was not permitted. Individual surgeons were not included in the randomization process and allocation concealment was ensured such that each center was required to call a designated, third-party representative before beginning the case. No changes in methods were attempted after trial commencement. All other data managers, patient interviewers, and principal investigator were blinded to assignments and randomization results were not released until the final patient had reached the primary and secondary endpoint time of follow-up.

### Device Implementation

The ECB is depicted in Fig. 1. The device was inserted after the patient was positioned but prior to draping and removed following the procedure prior to awakening from the patient. The device has two balloons: a smaller inner balloon that cycled 4 degrees saline continuously in order to cool the outer balloon. The outer balloon could be filled with up to 100 ccs of saline. In essence the device pulled the heat out of the surrounding local tissue. Surgeons could have the device partially or fully deflated if space issues became problematic. Cooling was initiated at the initiation of the surgical dissection. As noted, we previously demonstrated the cooling effect both by direct temperature measurement as well as MRI assessment of tissue cooling^21^.

### Outcome measurement

A third-party, independent statistician with extensive FDA trial experience was contracted to power and implement study design and randomization processes. A project medical director maintained all baseline demographic and follow-up data (including device randomization) at the coordinating center [University of California, Irvine]. At baseline, a history and physical exam included assessment of urinary and sexual function via American Urological Association Symptom Score (AUASS) and Urinary Quality of Life, and the International Index of Erectile Function – 5 (IIEF-5).

The primary and secondary outcomes of the study were improved pad-free continence at 90 days and a reduction in time to continence, respectively. There were no changes to trial outcomes after the trial commenced. Assessment of urinary continence was done both continuously and categorically: via questionnaires at 30, 60, 90, 180, and 365 days, and via pre-addressed continence postcards documenting three consecutive days of pad-free status. 90-day continence was a binary indicator of pad-free status at 90 days, while the secondary outcome measure of time to continence was continuously assessed. Patients were assessed for maintained continence at each time point; if a patient achieved continence at an early visit, he was still reassessed for continence at subsequent follow-up visits. During this one-year follow-up period, patients were ineligible for any incontinence intervention other than Kegel exercises. AUA symptom scores, urinary quality of life, urinary leakage, and IIEF-5 scores were also collected as part of routine follow-up. All follow-up continence data was collected by blinded third party contractors (one in Europe and one in the United States). Follow-up was required for one year. Statistical analysis on the clinical outcomes was performed by a second independent contractor.

### Statistical analysis

The sample size was based on level 0.05 (one-sided) test of equality of the probability of 90-day continence comparing the treatment to control. Based upon prior experience with the device (TA), it is expected that the probability of continence at 90-days post-surgery will be approximately 64%. Using this baseline probability, the current study would enroll a total of 180 patients (randomized 1:1 between treatment and control arm) to achieve 90% power for detecting an 18.8% increase in the probability of continence at 90-days post-surgery in the treatment arm as compared to the control arm. Maximum total accrual was set at 214 patients to allow for 10% dropout or loss to follow-up.

Independent variables included age (continuous and by age category by decade of life), race, height, and weight. Baseline disease characteristics included the American Urological Association (AUA) symptom score, the International Prostate Symptom Score (IPSS) bother question, prostate weight, and related co-morbidities. Independent variables were described and compared between groups with frequencies and proportions (the Pearson chi-square test), means, (student t-test), and medians (Wilcoxon 2-sample test).

A general linear model was used to estimate the difference in the probability of continence at 90 days with adjustment for the study site (included as a factored covariate). A 90% Wald-based confidence interval for the difference was also reported. Kaplan Meier analysis was used to analyze time to continence. Multivariate analysis was conducted by ranking in order of analysis and was tested using a hierarchical, step-down procedure for pairwise comparisons. This corresponds to a closed testing procedure and controls the overall level of significance at the two-sided, 0.10 level.

### Individual Surgeon Experience with the ECB device

The UroCool (Philips Healthcare Inc.) ECB device was used to achieve regional peri-prostatic hypothermia which had been demonstrated previously^19–21^. For those patients randomized to the hypothermia group, device insertion and use was the same as previously described^22^. The device was inserted and removed in the operating room while the patient was asleep. Knowledge of device use was restricted to the operating room personnel only; recovery room and ward nursing staff were not informed of device use and instructed to indicate such if a patient inquired. Patients otherwise received usual care associated with RARP.

Due to widely discordant surgeon continence outcomes at 90 days follow up, we analyzed each surgeon individually for ECB use. For each surgeon, the difference in probability of continence at 90 days and 1 year will be estimated and 90% Wald-based confidence intervals for the difference in each probability will be reported. Results were adjusted for covariates of age, BMI, PSA, AUA-SS and IIEF-5, all of which could affect return to continence.

Significance was considered at P < 0.05. Analysis was performed with SAS and final Kaplan Meier curves were designed in MedCalc.

## Results

### Baseline characteristics

**Figure 2:**
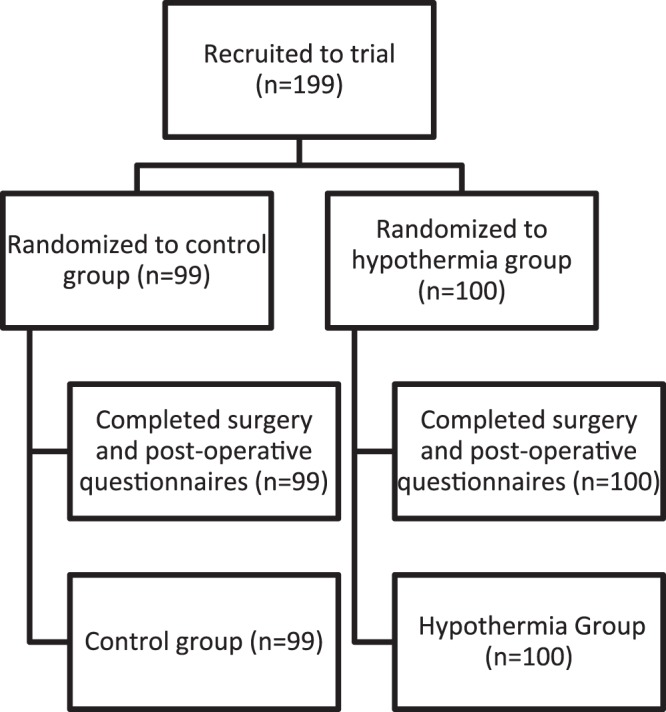
CONSORT Flow Diagram and Randomization.

Of the 199 patients who were recruited for the trial, 99 and 100 were randomized to the control and hypothermia groups, respectively. 100% of these patients completed pre- and postoperative interviews, received the intended treatment, and were analyzed for the primary and secondary outcome (Fig. 2). After recruitment from May through October 2013, one-year follow-up was obtained for all patients through November 2014, with no losses to follow-up or exclusions after randomization.

Baseline clinical characteristics were similar between the two groups, as presented in Table 1. At the time of RP, 16.0% versus 6.1% of patients in the hypothermia versus control groups had Gleason 8–10 disease (p = 0.143). The mean ± standard deviation in preoperative PSA was 8.79 ± 7.45 in the control and 8.19 ± 6.33 in the hypothermia group (p = 0.55). The degree of nerve-sparing was not different between the two groups. Of note, mean IIEF-5 scores were 17.1 versus 19.4 in the control versus hypothermia group (p < 0.03).

**Table 1:** Baseline demographics of the control versus experimental (ECB) arm.

	Control (n = 100)	UroCool (n = 99)	p-value
Age (mean years)	62.8	62.5	0.756
BMI (mean)	27.6	28.0	0.452
PSA (mean)	8.79	8.19	0.555
Pathologic Gleason Score			0.143
<3 + 3	1	1	
3 + 3	33	34	
3 + 4	34	32	
4 + 3	15	27	
8–10	16	6	
AUA SS (mean)	7.0	6.6	0.627
Bother Score (mean)	1.5	1.7	0.48
IIEF 5 (mean)	17.1	19.4	**0.03**
Patients with Diabetes (%)	13.0	7.0	0.15
Patients currently smoking (%)	9.0	15.0	0.201

### Outcomes

**Figure 3:**
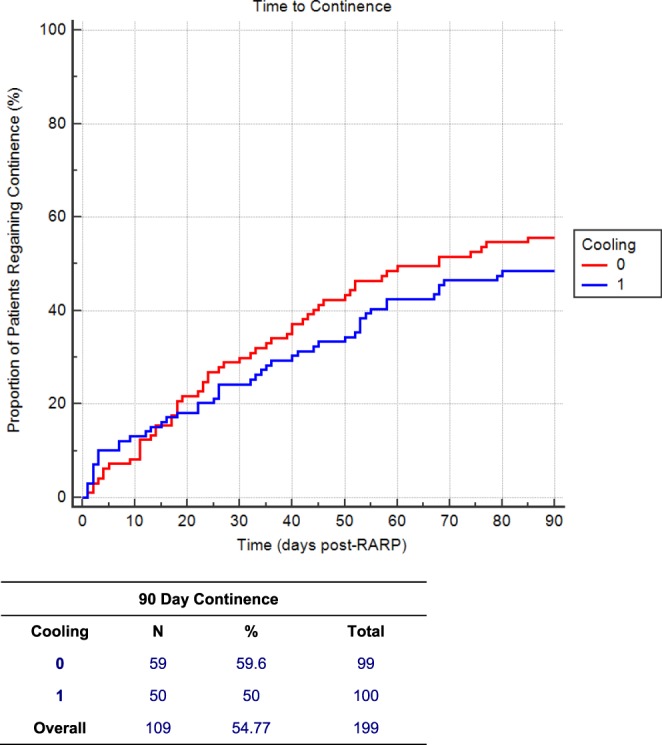
Time to 90-Day Continence between hypothermia and control group (p = 0.3242).

There were no adverse events (Clavien III and above) reported and trial follow-up ended after the final patient reached both the primary and secondary endpoint. Figure 3 depicts the primary and secondary outcome measures of dichotomous and time to 90-day pad-free continence. At 90-days, 59 (59.6%) and 50 (50.0%) patients in the control and hypothermia groups, respectively, had regained pad-free continence (p = 0.1748). Kaplan Meier analysis shows no statistically significant difference in time-to 90-day continence (p = 0.3242).

**Figure 4:**
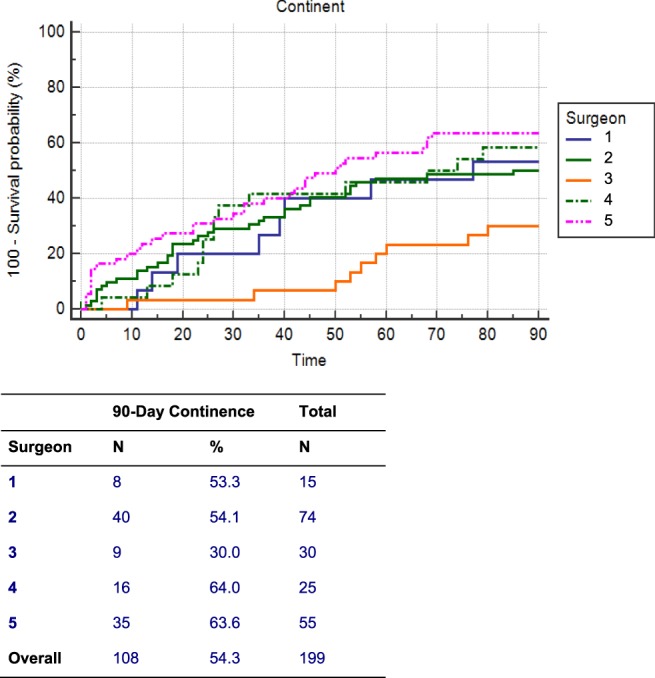
Time to 90-Day Continence between Surgeons.

When comparing time to continence by individual surgeon, the outcomes varied significantly at all follow-up time intervals. At 90-days of follow-up, individual surgeon continence rates varied from 30–64% when combining both hypothermia and control groups (Fig. 4). As randomization was conducted by the device, there was considerable variation between each surgeon as to the number of patients who did and did not receive hypothermia. Thus, unadjusted and adjusted analysis was performed to account for surgeon technique and patient-dependent covariates (Table 2). Although there was a higher continence rate at 30-days, it was not statistically significant. All other time points showed a negative effect, again, none which reached statistical significance.

**Table 2:** Multivariate cox proportional hazard model of continence controlling for covariates (BMI, PSA, AUA-SS, IIEF-5, Surgeon).

	OR	95% CI		p-value
		Lower	Upper	
30 Days	1.406	0.666	2.968	0.372
60 Days	0.668	0.359	1.244	0.203
90 Days	0.750	0.412	1.364	0.345
6 Months	0.746	0.379	1.470	0.398

Lastly, each of the individual surgeons underwent exploratory adjusted analysis for ECB benefit (data not shown); one surgeon had an overall trend of a non-significant, positive effect whilst the rest of the surgeons had non-significant, negative impact. Individual surgeon experience and outcomes were analyzed according to ECB use. Time to continence by surgeon in controls, hypothermia, and overall patient cohort significantly varied. Hypothermia versus controls showed no statistically significant differences in continence recovery in any of the five surgeons.

## Discussion

Despite our initial findings supporting an anti-inflammatory benefit from regional hypothermia with a reduction in median time to pad-free continence and improved overall pad-free continence^22^, this multi-institutional randomized control trial with five high-volume surgeons did not validate the use of this device. Figure 3 demonstrates that the presence of the device did not improve the primary outcome of categorical 90 day pad-free continence or the secondary outcome of Kaplan Meier time-to 90 day pad-free continence. The assumption at the outset of the trial was that a high volume would minimize the impact of surgeon outcome variability. However, Fig. 4 demonstrates extensive variation in continence rates among the five surgeons - as much as 10-fold. This variation in continence rates are consistent with previous trials depicting functional outcomes post-robot-assisted RP^23,24^.

An interesting consideration of this trial is whether hypothermia might work for some surgeons and not for others. In the initial study, having a single surgeon perform all the procedures may have eliminated a significant confounding factor in the present study: inter-surgeon outcome variability. After performing adjusted analysis, it appears that one surgeon experienced a trend towards improved continence. The remaining surgeons, however, demonstrated no evidence of benefit in unadjusted or adjusted analysis.

The results are hypothesis-generating. Theoretically, one could reason that a less-skilled surgeon who creates more trauma could have shown the greatest benefit in hypothermia use. This however was not demonstrated. There are a couple of hypotheses that could explain these findings: although it is common knowledge that cooling a torn ligament will typically heal it more quickly, the cooling of a ruptured ligament would relieve pain but would have no mechanism for faster healing. This also suggests that not inducing surgical trauma or inflammation is (much) more effective than accepting the trauma and trying to preemptively prevent the inflammatory process with hypothermia. While past experience with regional hypothermia confirmed an advantage in the single-surgeon setting, the variability of surgical technique and skill in this study suggests that resources would be more effectively devoted to decreasing surgical trauma. A similar finding may be suggested from the study of preventing renal injury during partial nephrectomy^25^. Historically, it is well established that permanent renal dysfunction due to purely ischemic inflammatory injury can be dramatically reduced with regional hypothermia. However, in the present setting, we had not only ischemic issues but also traumatic injury that potentially nullifying any benefit associated with cooling.

The results also highlight the limitations and inherent challenges of conducting randomized control trials (RCTs) in the context of surgery. Importantly, several articles have noted difficulties of conducting RCTs of surgical interventions^26–29^. RCTs have been seen as the gold standard in medical research due to their ability to eliminate selection bias when conducted correctly. RCTs are designed to control for one confounder and any surgical intervention is potentially susceptible to multiple confounders such as device-, surgeon- and patient- dependent characteristics^24,30^. Using our initial power calculation, the significant difference between surgeons would have required 200 patients to be randomized per surgeon to potentially discern relevance of individual surgeons. The outcomes of this randomized control trial strongly suggest that regional hypothermia would add no benefit to the majority of surgeons and resources would be more effectively devoted to improving surgical skill.

## Conclusion

Although background and preliminary data showed regional hypothermia with ECB had the scientific and clinical potential to be beneficial in improving return to continence, this was not demonstrated in the present RCT.

### Ethics approval and consent to participate

The institutional review board (IRB) of each center approved the study, compliance with the Health Insurance Portability and Accountability was maintained, and the trial was formally registered as a randomized control trial. The following IRBs reviewed the protocols at their respective institutions:

- Ethik-Kommission der Ärztekammer Westfalen-Lippe und der Westfälischen Wilhelms-Universität Münster (CG, JW).
- City of Hope National Medical Institutional Review Board (TW, CL).
- Western Institutional Review Board (JP).

## Data Availability

The datasets generated and/or analysed during the current study are not publicly available due to the Health Insurance Protability and Accountability Act. The data is available from the corresponding author on reasonable request and documented institutional review board approval and/or data use agreements.
